# Pragmatic Emergency Department Intervention Reducing Default Quantity of Opioid Tablets Prescribed

**DOI:** 10.5811/westjem.18040

**Published:** 2024-05-20

**Authors:** Drake Gotham Johnson, Alice Y. Lu, Georgia A. Kirn, Kai Trepka, Yesenia Ayana Day, Stephen C. Yang, Juan Carlos C. Montoy, Marianne A. Juarez

**Affiliations:** University of California San Francisco, Department of Emergency Medicine, San Francisco, California; °Johnson, Lu, Kirn, Trepka, Day, and Yang are co-first authors.

## Abstract

**Introduction:**

The opioid epidemic is a major cause of morbidity and mortality in the United States. Prior work has shown that emergency department (ED) opioid prescribing can increase the incidence of opioid use disorder in a dose-dependent manner, and systemic changes that decrease default quantity of discharge opioid tablets in the electronic health record (EHR) can impact prescribing practices. However, ED leadership may be interested in the impact of communication around the intervention as well as whether the intervention may differentially impact different types of clinicians (physicians, physician assistants [PA], and nurse practitioners). We implemented and evaluated a quality improvement intervention of an announced decrease in EHR default quantities of commonly prescribed opioids at a large, academic, urban, tertiary-care ED.

**Methods:**

We gathered EHR data on all ED discharges with opioid prescriptions from January 1, 2019–December 6, 2021, including chief complaint, clinician, and opioid prescription details. Data was captured and analyzed on a monthly basis throughout this time period. On March 29, 2021, we implemented an announced decrease in EHR default dispense quantities from 20 tablets to 12 tablets for commonly prescribed opioids. We measured pre- and post-intervention quantities of opioid tablets prescribed per discharge receiving opioids, distribution by patient demographics, and inter-clinician variability in prescribing behavior.

**Results:**

The EHR change was associated with a 14% decrease in quantity of opioid tablets per discharge receiving opioids, from 14 to 12 tablets (*P* = <.001). We found no statistically significant disparities in prescriptions based on self-reported patient race (*P* = 0.68) or gender (*P* = 0.65). Nurse practitioners and PAs prescribed more opioids per encounter than physicians on average and had a statistically significant decrease in opioid prescriptions associated with the EHR change. Physicians had a lesser but still significant drop in opioid prescribing in the post-intervention period.

**Conclusion:**

Decreasing EHR defaults is a robust, simple tool for decreasing opioid prescriptions, with potential for implementation in the 42% of EDs nationwide that have defaults exceeding the recommended 12-tablet supply. Considering significant inter-clinician variability, future interventions to decrease opioid prescriptions should examine the effects of combining EHR default changes with targeted interventions for clinician groups or individual clinicians.

Population Health Research Capsule What do we already know about this issue?

*Emergency department opioid prescriptions increase the incidence of opioid use disorder in a dose-dependent manner, potentially exacerbating the opioid epidemic.*

What was the research question?

*This study evaluated the impact of a quality improvement intervention decreasing default opioid quantities in the EHR from 20 pills to 12, on average opioids prescribed at discharge.*

What was the major finding of the study?

*The EHR change was associated with a 14% decrease in quantity of opioid tablets per discharge receiving opioids (P < .001), driven mostly by nurse practitioners’ and physician assistants’ changes.*

How does this improve population health?

*We demonstrate a simple intervention other emergency departments can immediately implement to reduce the burden opioid prescribing has on the opioid epidemic.*

## INTRODUCTION

The opioid epidemic is a major cause of morbidity and mortality in the United States, including in California.^1^ Opioid prescriptions initiated in the emergency department (ED) and other clinical care settings can increase the incidence of opioid use disorder (OUD) in a dose-dependent manner—the more tablets prescribed, the greater the risk of future development of OUD.^2^^–^^4^ In addition, the presence of excess opioid tablets in the home is linked to diversion and overdose.^5^ Decreasing the total quantity of tablets prescribed from the ED may help decrease the risk of these harms.

Many interventions attempt to decrease and alleviate the risks of opioid prescriptions in ED settings, from electronic clinical decision support alerts to co-prescription of naloxone, but most existing ED interventions focus on decreasing prescription rates rather than decreasing the quantity of opioid tablets prescribed when ED patients are discharged with opioids.^6^^–^^8^ Prior research has shown that decreasing the default quantity of tablets prescribed in the electronic health record (EHR) without announcing the change to clinicians can decrease the number of opioids per prescription given at discharge. In these studies, clinicians were not notified of altered EHR default prescriptions either for convenience or to test the effect of a default change alone, or due to concern that clinicians would consciously override the defaults.^9^^–^^13^

Because protocol changes in the ED are commonly arrived at by consensus and are usually implemented transparently rather than unannounced, studying the effect of an announced EHR change more closely mirrors real-world scenarios. An announcement about the change may have the added benefit of educating clinicians about opioid prescribing guidelines, the risks of prescribing opioids, and signals what other clinicians are thinking about opioid prescriptions. Further, there is evidence that nurse practitioners (NP) and physician assistants (PA) are more likely than physicians to prescribe opioids in primary care settings,^14^ but the relationship between clinician type and opioid-prescribing behavior in the ED setting remains unknown. In addition, prior work has not shown whether these different types of clinicians respond similarly to default-directed attempts to decrease opioid prescribing.

To address these gaps, we implemented a quality improvement (QI) intervention decreasing EHR default quantities of commonly prescribed opioids at a large, academic, urban, tertiary-care center. Our goal was to determine whether this EHR change was associated with decreased opioid prescribing and whether this association varied by clinician type.

## METHODS

### Design

We implemented a single-site, QI intervention at a large, academic, urban tertiary-care ED altering the default quantity of six commonly prescribed opioids. This was a prospective QI study where data was pulled from chart review and analyzed both during study design and continuously during implementation. We collected pre-intervention data on all ED discharges receiving these six opioids at discharge from January 1, 2019–March 28, 2021, and compared this with post-intervention data from March 29, 2021–December 5, 2021. This work was considered QI activity according to the University of California, San Francisco institutional review board policy. As a result, the requirement for individual research HIPAA authorization and signed consent forms was waived for all subjects as the research presented no more than minimal risk of harm to the subjects’ privacy.

### Intervention

We decreased the pre-populated ED discharge dispense quantities in the EHR from 20 tablets to 12 tablets for the following six commonly prescribed opioids: oxycodone 5 milligrams (mg); oxycodone-acetaminophen 5–325 mg; oxycodone 10 mg; tramadol 50 mg; hydrocodone-acetaminophen 5–325 mg; and hydrocodone-acetaminophen 10–325 mg. Changes were made at the system level and applied to all ED patients and clinicians. Clinicians decided for whom to prescribe opioids and could choose any quantity by altering the default setting. Clinicians in the ED were informed of the study and quantity changes using two communication methods: by two email announcements sent to all physicians, PAs, and NPs; and by two in-person announcements during the weekly all-staff ED meetings attended by 10–12 total physicians, PAs, and NPs. The email and weekly all-staff announcements were made over a period of two weeks prior to the intervention.

### Participants

We included ED patient encounters in which patients were discharged from the ED with a prescription for one of the six opioid medications included in the intervention. We also recorded the total number of patients discharged from the ED each month during the period of our study, regardless of whether they were given a prescription at the end of their visit. Each encounter was recorded as an observation, regardless of whether these patients had other ED visits.

### Outcomes

From all ED encounters that had an opioid medication prescribed at discharge, we extracted the following data from the EHR: date of visit; patient demographics (race, age, gender, insurance type); acuity (based on the assigned Emergency Severity Index score in the EHR), chief complaint, prescribing clinician type, opioid medication prescribed and quantity of tablets. Insurance type was categorized as Medicaid, Medicare, commercial, self-pay, or other. Chief complaints were classified into the four most common chief complaints seen in our ED over the study period (back pain, abdominal pain, flank pain, falls), with the remaining chief complaints grouped as “other.” Prescribing clinician types were categorized as physician, NP, or PA.

Our primary outcome measure was the difference in mean number of opioid tablets prescribed at discharge before and after our intervention. Our secondary outcomes included differences in this measure given the patient’s self-reported race and self-reported gender, as well as prescribing clinician type for the encounter (physician, NP, PA). We also tested the difference in mean morphine milligram equivalents (MME) prescribed at discharge before and after our intervention.

### Analysis

We calculated MMEs using the conversion factors provided by the US Centers for Disease Control and Prevention (CDC).^4^ Frequency tables were generated for categorical variables. Median and interquartile range were generated for age and means, and standard deviations were calculated for all other continuous variables. We performed two sample *t*-tests to compare mean opioid tablets prescribed before and after our intervention and calculated 95% confidence intervals (CI). Given the effect of the COVID-19 pandemic on the volume of ED discharges during our pre-intervention data collection, we performed sensitivity analyses restricting the study period to different start times, including after the start of the COVID-19 pandemic (in March 2020). We performed chi-square tests of independence for age, race, insurance type, and acuity before and after intervention, and the Fisher exact test for gender. Two-way analysis of variance (ANOVA) was performed to analyze the interaction between clinician type and intervention on mean opioid tablets prescribed. *P* values < 0.05 were reported as significant. We performed all analyses using Python 3 (Python Software Foundation, Wilmington, DE).

## RESULTS

There were 3,575 ED discharges with an opioid prescribed during the study period, of which 3,274 (91.6%) had prescriptions for one of the six opioids targeted by our intervention, including 2,666 discharges pre-intervention and 608 discharges post-intervention. **Opioids not targeted** by our intervention included morphine (2.5%), hydromorphone (1.4%), **oxycodone** (1.3%), hydrocodone (<1%), codeine (<1%), **tramadol** (<1%), methadone (<1%), and fentanyl (<1%). The patient population seen in the ED pre- and post-intervention had similar distributions of discharge diagnoses, age, gender, self-reported race, acuity, insurance type, and prescribing clinician type (Table 1). There were no statistically significant differences in prescriptions between individuals with different self-reported races (chi-squared *P* = 0.68) or between genders (Fisher exact *P* = 0.65) before and after implementation of our intervention.

**Table 1.** Patient demographics of opioid prescriptions in the emergency department.

Patient demographics	All	Pre	Post	*P* value
Age, median (IQR)	48 (27)	48 (27)	48 (29)	0.88
Gender, n (%)				0.65
Female	1,707 (0.522)	1,395 (0.5242)	312 (0.514)	
Male	1,561 (0.478)	1,266 (0.4758)	295 (0.486)	
Race, n (%)				0.69
White	1,719 (0.525)	1,393 (0.5225)	326 (0.536)	
Black	423 (0.129)	353 (0.1324)	70 (0.115)	
Asian	467 (0.143)	382 (0.1433)	85 (0.14)	
Other	665 (0.203)	538 (0.2018)	127 (0.209)	
Acuity, n (%)				0.29
Emergent	286 (0.087)	243 (0.0912)	43 (0.071)	
Urgent	2,013 (0.615)	1,618 (0.6071)	395 (0.65)	
Less urgent	947 (0.289)	781 (0.2931)	166 (0.273)	
Non-urgent	27 (0.008)	23 (0.0086)	4 (0.007)	
Insurance, n (%)				0.53
Commercial	1,448 (0.442)	1,172 (0.4396)	276 (0.454)	
Medicaid	801 (0.245)	662 (0.2483)	139 (0.229)	
Medicare	702 (0.214)	571 (0.2142)	131 (0.216)	
Self-pay	167 (0.051)	140 (0.0525)	35 (0.058)	
Other	156 (0.048)	121 (0.0454)	27 (0.044)	
Clinician, n (%)				0.42
Physician	1,573 (0.481)	1,269 (0.476)	304 (0.5)	
NP	862 (0.263)	714 (0.268)	148 (0.243)	
PA	839 (0.256)	683 (0.256)	156 (0.257)	
Discharge diagnosis, n (%)				0.38
Abdominal pain	425 (0.130)	345 (0.129)	80 (0.131)	
Back pain	324 (0.0990)	258 (0.0968)	66 (0.109)	
Flank pain	292 (0.0892)	248 (0.0930)	44 (0.0724)	
Fall	190 (0.0580)	41 (0.0559)	149 (0.0674)	
Other	2,043 (0.624)	1,666 (0.624)	377 (0.620)	

*IQR*, interquartile range; *NP*, nurse practitioner; *PA*, physician assistant.

**Figure 1.**
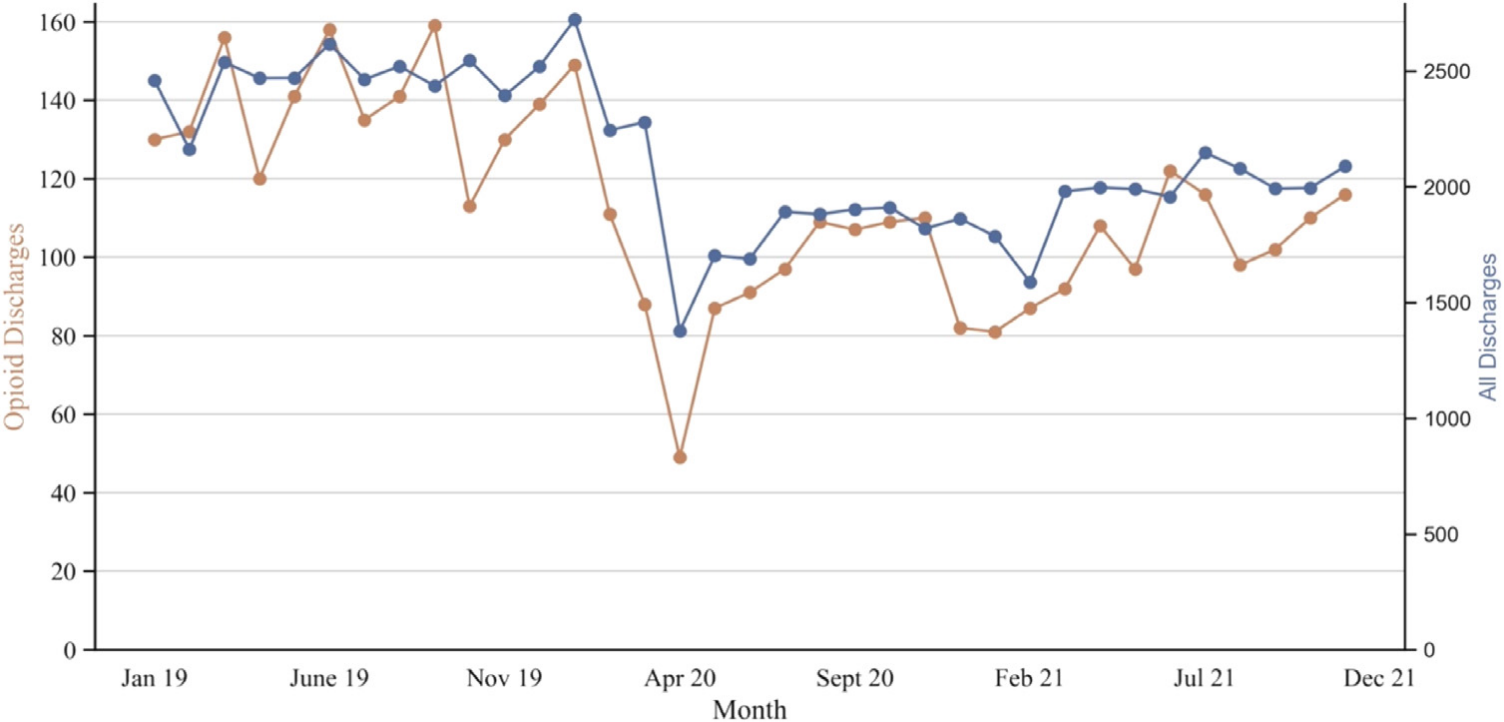
Decreasing default opioid quantities in the electronic health record is associated with lower ED prescription of opioids in the emergency department. Number of total discharges (blue) and discharges in which opioids were prescribed (orange) over the study timeline. The intervention began on March 19, 2021.

The number of ED encounters associated with an opioid prescription upon discharge was proportional to the total number of discharges from the ED throughout the study period, although both experienced a precipitous decline at the start of the COVID-19 pandemic (Figure 1).

Decreasing the EHR default quantity of commonly prescribed opioids was associated with a decrease from 14.01 to 12.00 tablets per discharge prescription with opioids from the ED, a difference of 2.01 tablets (95% CI 1.44–2.58) (Table 2). Sensitivity analysis showed there was a statistically significant difference in tablets prescribed regardless of how many months were included in the pre-intervention dataset (Supplemental Table 1). This decrease in tablets is mirrored by an 11.0 MME decrease per discharge prescription with opioids (95% CI 5.74–16.22) from 94.25 to 83.27 (Table 2).

**Table 2.** Tablets and morphine milligram equivalents per discharge prescription with opioids.

Opioid prescriptions	All	Pre	Post	Δ (95% CI)	*P* value
	Mean (SD)	Mean (SD)	Mean (SD)		
Tablets per opioid discharge	13.63 (6.54)	14.01 (6.75)	12.00 (5.22)	−2.01	<.001
				(−2.58, −1.44)	
MME per opioid discharge	92.21 (59.60)	94.25 (62.18)	83.27 (45.60)	−11.0	<.001
				(−16.22, −5.74)	

*CI*, confidence interval; *MME*, morphine milligram equivalent.

**Figure 2.**
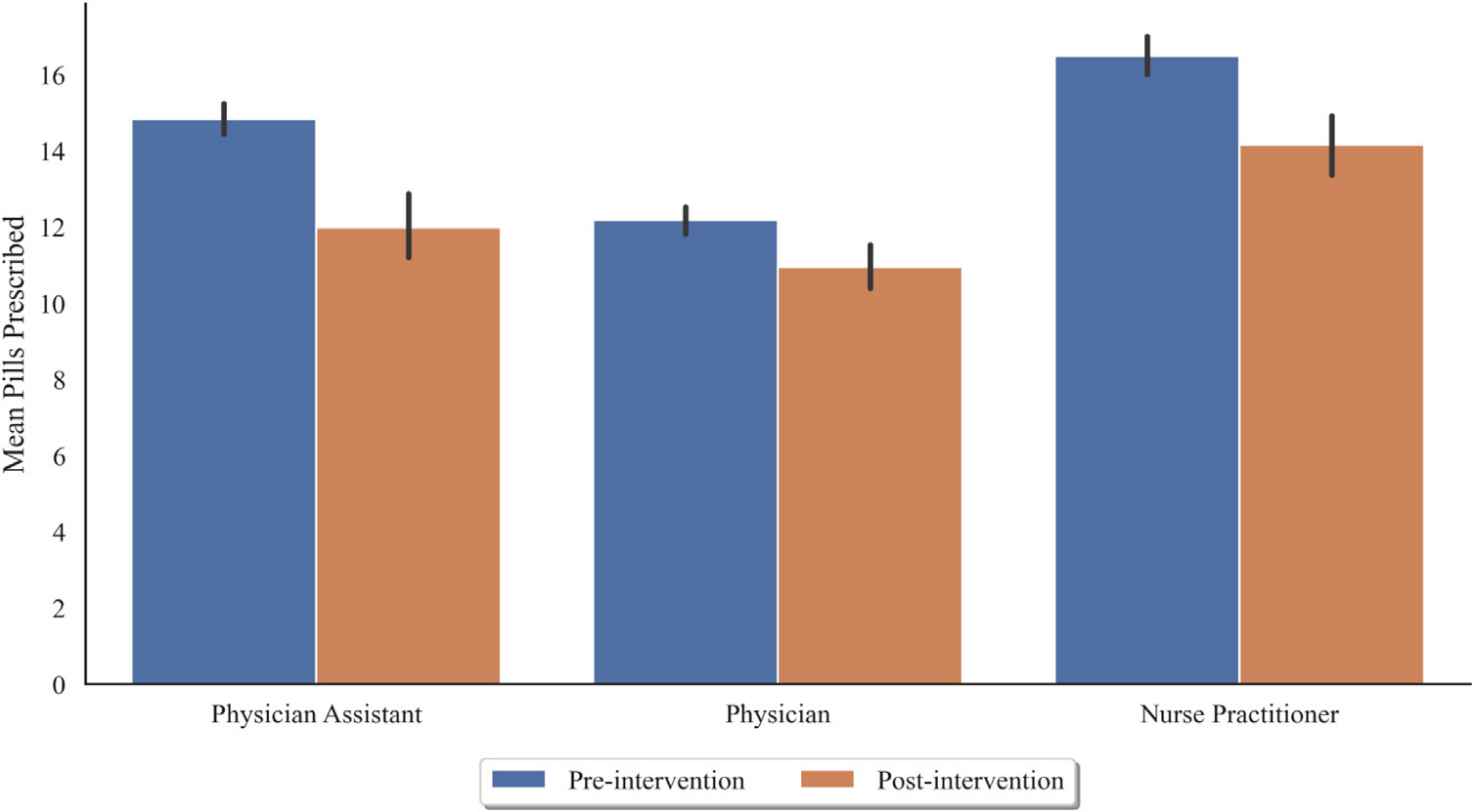
Clinician type is associated with opioid prescription quantities in the emergency department. Average number of tablets per discharge in which opioids were prescribed, grouped by clinician type and intervention time (blue = pre-intervention, orange = post-intervention).

For 2,666 pre-intervention encounters in the dataset, physicians wrote 47.6% of study prescriptions, NPs wrote 26.8%, and PAs wrote 25.6% of study prescriptions. For the 608 post-intervention encounters in the dataset, physicians wrote 50% of study prescriptions, NPs wrote 24.3%, and PAs wrote 25.7% of study prescriptions. All clinician types prescribed significantly fewer opioids per encounter after the intervention compared to prior, with PAs and NPs affected the most (Figure 2, Table 3). A two-way ANOVA of the clinician type and intervention confirmed statistically significant effects of the intervention, clinician type, and interaction between intervention and clinician type on the number of tablets per discharge prescription with opioids (*P* < 0.001).

**Table 3.** Number of tablets per discharge prescription with opioids, by clinician type.

Clinician type	All	Pre	Post	Δ (Post minus Pre)	95% CI
NP	16.09	16.49	14.16	−2.33	(−3.40, −1.08)
Physician	11.93	12.17	10.95	−1.22	(−2.08, −0.46)
PA	14.30	14.83	11.99	−2.84	(−3.80, −1.88)

*NP*, nurse practitioner; *PA*, physician assistant; *CI*, confidence interval.

## DISCUSSION

We implemented an announced decrease in EHR default quantities of six commonly prescribed opioids at a large, academic, urban, tertiary-care ED. The analysis of our primary outcome showed that this QI intervention was associated with a statistically significant decrease in opioid tablets per discharge prescription with opioids from the ED, from 14 to 12 tablets, and a corresponding 11-point decrease in mean MMEs prescribed. While no studies have precisely quantified the clinical significance of this level of decrease, prior literature and CDC guidelines note a dose-dependent relationship between prescriptions and risk of developing OUD, suggesting that every pill matters at a population level.^2^^–^^4^ Further, given that this center’s pre-intervention mean tablets per ED discharge opioid prescription was only 14, the maximum expected decrease from a default change to 12 was only a decrease of two tablets per discharge prescription. However, these interventions might confer a larger clinical significance at other institutions with a higher starting mean tablets per discharge. Importantly, we observed that NPs and PAs in the ED setting are more likely than physicians to prescribe higher levels of opioids at baseline, consistent with previous results in primary care settings.^14^

Our results suggest that a universal default change is associated with decreased opioid prescriptions across all clinicians, with larger decreases for NPs and PAs compared to the change observed for physicians. The higher rates of opioid prescriptions among NPs and PAs could be due to a variety of factors, including differences in the acuity or types of illnesses and injuries evaluated. Additionally, even after the intervention, the high average opioids prescribed in the NP group was driven by a few clinicians still far exceeding the default (Supplemental Figure 1). The existence of inter-clinician variability in prescriptions may provide opportunities for more targeted future interventions, such as NP- or PA-specific interventions in conjunction with EHR-driven interventions.

We chose to analyze the average number of tablets prescribed per encounter in which opioids were prescribed rather than per ED visit or per month. Average number of tablets aligns more directly with our intervention, which was aimed at reducing the quantity of opioids prescribed after a clinician had already determined a need for opioid analgesia. Additionally, the number of tablets prescribed per opioid encounter is less impacted by temporal and seasonal variation in prescribing patterns and visit acuity, including the effect of the COVID-19 pandemic.

In most prior studies, clinicians were not notified of altered EHR default prescriptions either for convenience or to test the effect of a default change alone, or due to concern that clinicians would consciously override the defaults.^9^^–^^13^ However, we found that decreasing default EHR opioid quantities to 12 tablets coupled with informing clinicians of the EHR change resulted in a decrease in the total number of opioids prescribed at ED discharge. We observed decreases in the average number of tablets prescribed per patient and the average MME of tablets prescribed per patient. This suggests that transparency with clinicians regarding best practices in opioid prescribing does not negate the effect of altering EHR defaults. It is possible that an announcement to clinicians about the EHR change and the rationale behind it may serve as an educational feedback component to the intervention. Clinicians who appreciate the purpose of the default change may be more likely to use the default, go lower than the default, or even write fewer prescriptions as they see fit for each clinical scenario, consistent with prior work demonstrating that audit and feedback approaches can decrease opioid prescribing.^15^

Because prior work has demonstrated the existence of racial disparities in opioid prescribing, we investigated whether clinicians’ opioid prescribing behavior differed based on patient demographics.^16^ Our analysis showed that there was no statistically significant disparity in opioid prescription amounts based on patient demographics, including age, race, and gender, for both the pre- and post-intervention data.

It is also important to note that the COVID-19 pandemic started during our pre-intervention phase, which resulted in an overall decrease in ED utilization.^17^ However, our outcome is somewhat insulated from changes in ED volume, as tablets per prescription should not be dependent on the number of patient discharges. The COVID-19 pandemic may have led to other more subtle changes in prescribing behavior secondary to changing patient populations seen, but the major chief complaints did not differ in the pre- and post-intervention period, and the results of our sensitivity analysis confirmed that the effect seen was still present even after restricting our data to an entirely post-COVID-19 timeframe.

Ultimately, we recognize that opioids remain first-line treatments for certain indications such as short-term pain relief for acute fractures and cancer pain and are often necessary at discharge from the ED. However, given the risks of diversion, overdose, and OUD associated with discharging patients with large quantities of opioid tablets, it is important to encourage emergency clinicians to discharge patients with a clinically appropriate yet safe quantity of tablets. It is also important to use discretion as opioids are often not indicated for certain other causes of pain in patients presenting to the ED, including the common chief complaints of abdominal pain and lower back pain.^18^ Recommendations for acute pain suggest discharging patients with a three-day supply of opioid medications, which corresponds to 12 tablets or less.^19^ Our approach is a pragmatic, transparent, and scalable intervention that offers a tool that can be implemented in the 42% of EDs nationwide that currently have defaults exceeding 12 tablets.^19^

## LIMITATIONS

Our study design of a single-site, pre/post study does not allow for a causal interpretation and limits generalizability. Much of the project occurred during the COVID-19 pandemic, in which opioid prescribing increased nationwide; however, patterns for ED discharge prescriptions have not been studied.^20^ Our design did not allow us to measure associated harms or benefits, such as whether pain control was adequate or whether diversion decreased.^21^ Neither did our design allow us to test for differences in whether patients were prescribed opioids, which is also an important consideration for opioid stewardship. Additionally, the 12-tablet default quantity was chosen to approximate a three-day supply, but this length may vary based on the frequency prescribed of a given opioid, and there is limited evidence to support the optimal time course of opioids at discharge.^22^

Finally, the study design did not allow us to measure the precise number of clinicians who were exposed to the clinician-facing announcement, differentiate whether the effects observed were attributable to the EHR changes alone, the clinician-facing announcement alone, or a combination of the two.

## CONCLUSION

We demonstrated that a quality improvement intervention coupling decreased default opioid quantities in the electronic health record with informing clinicians of the EHR change was associated with a decrease in the total number of opioids prescribed from the ED. While all clinician types (NPs, PAs, and physicians) decreased their quantities of opioids prescribed per discharge following the default change, NPs and PAs prescribed more opioids than physicians initially and experienced a larger decrease in opioid prescriptions. Future interventions seeking to address ED opioid prescribing should measure the total quantity of opioids leaving the ED over longer periods of time, use a robust, patient-centered metric for pain management follow-up, and attempt to correlate ED opioid prescriptions with negative opioid-associated outcomes in both individual patients and their communities.

## Supplementary Information



