# Supplementary material for: Pragmatic Emergency Department Intervention Reducing Default Quantity of Opioid Tablets Prescribed
Source: West J Emerg Med. 2024 May 20;25(4):449–56. doi: 10.5811/westjem.18040 (PMC11254152; doi:10.5811/westjem.18040)
Supplement: Supplementary file 1 [file wjem-25-449-s001.docx]

**Supplemental Table 1:** Sensitivity analysis

| **Start month** | **Total days pre intervention** | **P value for primary outcome** |
| --- | --- | --- |
| 2019-01-31 | 788 days | <0.0001 |
| 2019-02-28 | 760 days | <0.0001 |
| 2019-03-31 | 729 days | <0.0001 |
| 2019-04-30 | 699 days | <0.0001 |
| 2019-05-31 | 668 days | <0.0001 |
| 2019-06-30 | 638 days | <0.0001 |
| 2019-07-31 | 607 days | <0.0001 |
| 2019-08-31 | 576 days | <0.0001 |
| 2019-09-30 | 546 days | <0.0001 |
| 2019-10-31 | 515 days | <0.0001 |
| 2019-11-30 | 485 days | <0.0001 |
| 2019-12-31 | 454 days | <0.0001 |
| 2020-01-31 | 423 days | <0.0001 |
| 2020-02-29 | 394 days | <0.0001 |
| 2020-03-31 | 363 days | <0.0001 |
| 2020-04-30 | 333 days | <0.0001 |
| 2020-05-31 | 302 days | <0.0001 |
| 2020-06-30 | 272 days | <0.0001 |
| 2020-07-31 | 241 days | <0.0001 |
| 2020-08-31 | 210 days | <0.0001 |
| 2020-09-30 | 180 days | <0.0001 |
| 2020-10-31 | 149 days | 0.00076 |
| 2020-11-30 | 119 days | 0.0071 |
| 2020-12-31 | 88 days | 0.0056 |
| 2021-01-31 | 57 days | 0.022 |
| 2021-02-28 | 29 days | 0.012 |
