# Supplementary figures and images for: Pragmatic Emergency Department Intervention Reducing Default Quantity of Opioid Tablets Prescribed
Source: West J Emerg Med. 2024 May 20;25(4):449–56. doi: 10.5811/westjem.18040 (PMC11254152; doi:10.5811/westjem.18040)

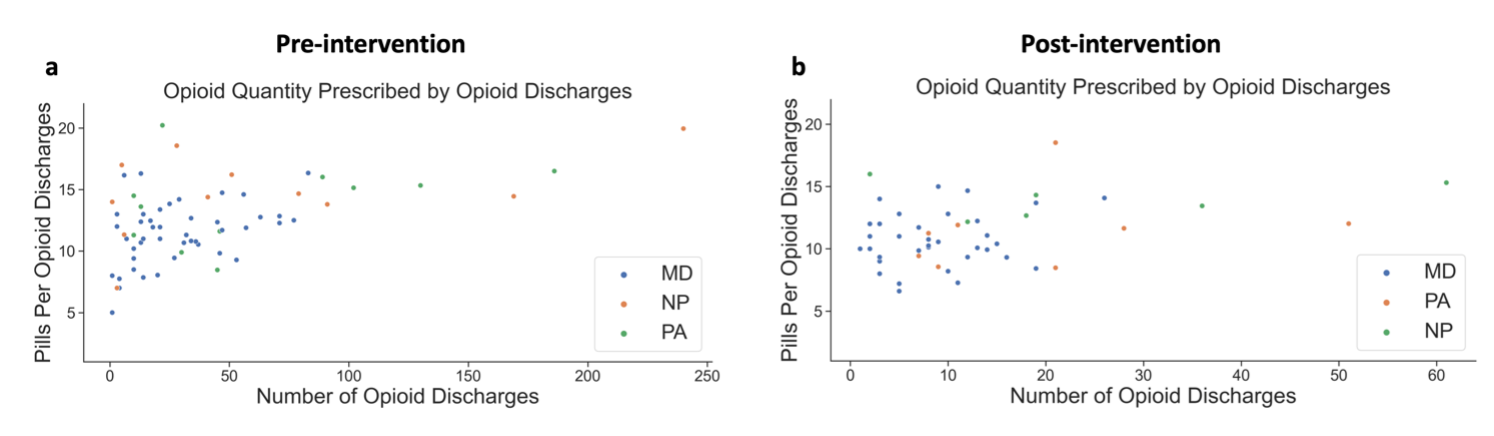

Supplement: Supplementary file 2 [file wjem-25-449-s002.png]
